# Factors controlling accumulation of organic carbon in a rift-lake, Oligocene Vietnam

**DOI:** 10.1038/s41598-020-71829-7

**Published:** 2020-09-11

**Authors:** M. Rizzi, J. Hovikoski, N. H. Schovsbo, J. Therkelsen, M. Olivarius, H. P. Nytoft, L. H. Nga, N. T. T. Thuy, D. M. Toan, J. Bojesen-Koefoed, H. I. Petersen, L. H. Nielsen, I. Abatzis, C. Korte, M. B. W. Fyhn

**Affiliations:** 1 Department of Geosciences and Natural Resource Management, University of Copenhagen, Øster Voldgade 10, 1350 Copenhagen, Denmark; 2 Geological Survey of Denmark and Greenland, Øster Voldgade 10, 1350 Copenhagen, Denmark; 3 Exploration & Production Centre, Vietnam Petroleum Institute, 167 Trung Kinh, Yen Hoa, Cau Giay, Hanoi, Vietnam; 4 Total Upstream Danmark A/S, Amerika Plads 29, 2100 Copenhagen, Denmark

**Keywords:** Biogeochemistry, Climate sciences, Limnology

## Abstract

Understanding of the processes of petroleum source rock (SR) accumulation in lacustrine rift basins and the behavior of lake systems as long-term carbon sinks is fragmentary. Investigation of an 800 m thick (500 m core and ~ 300 m outcrop), deep-lacustrine, Oligocene section in Vietnam, provides a rare insight into the controls and deposition of organic carbon (OC) and SR formation in continental rift basins. A multidisciplinary dataset, combining elemental data, inorganic and organic geochemistry with sedimentology, shows that the richest alginite-prone, sapropelic SR developed during periods of relative tectonic quiescence characterized by moderate primary productivity in a mainly dysoxic lacustrine basin. Increased rift activity and further development of graben morphology intensified water column stratification and anoxia, which hindered nutrient recycling. Sapropelic organic matter (OM) continued to accumulate, but with increasing amorphous OM content and decreasing total OC values. Periods of increased seasonality were characterized by thermocline weakening, enhanced mixing of water columns, increased primary productivity and diatom blooming. The results suggest that a change from dysoxia towards anoxia or extreme primary productivity does not necessarily enhance OC burial and SR quality. External nutrient input from a phosphate-rich hinterland is sufficient for sapropel formation, whereas the main limiting factor is methanogenesis.

## Introduction

Organic carbon (OC) accumulation and development of petroleum source rocks (SR) are complex processes involving a number of interrelated factors, the relative importance of which are not fully understood^1,2^. Furthermore, many of the factors can be scale dependent^3^ what makes direct comparison with studies dealing with surface sediments, representing shorter time spans or different settings convoluted^4–7^. Lacustrine rift basins are especially challenging in this regard due to localized and evolving topography through the active rifting phase, which typically cause profound changes in factors controlling organic matter (OM) production and preservation^8^. Consequently, predicting OM quantity and quality is typically complicated in such settings, when geological verification is not possible. Unfortunately, rift lake SR intervals are rarely penetrated by drill cores, limiting our understanding of SR development in such systems as well as their behavior as long-term carbon sinks.

Significant insights into OC accumulation in lacustrine rift basin have been derived particularly from the modern East African lakes^6,7,9–11^. These studies have revealed the importance of factors such as width-depth ratio on the lake’s mixing potential, primary productivity and anoxia^10^. In addition to internal nutrient recycling, hinterland derived nutrient supply can be important for productivity, and depend on evolution of the hinterland topography^3,12^. Several studies have also documented the sensitivity of lake levels for high-frequency climatic changes^5,11,13^, a feature that is less straightforward to reconstruct from ancient sediment series. Despite of some contrasting views of controlling factors, the data generally indicate that tropical rift lakes form an ideal setting for OM and SR accumulation^6,14^.

The controlling factors behind OM and SR accumulation in an Oligocene, low-latitude, deep rift lake system in Vietnam are investigated by integrating handheld X-ray fluorescent (HH-XRF) analyses, source rock screening and sedimentological data, supported by scanning electron microscope (SEM), selected biomarkers and maceral analyses. The results allow an assessment of the tectonic and climatic (seasonality) controls on sedimentation, broad scale changes in water depth, evolution of rift basin morphology, changes in water column oxygenation and thermocline, effects of diagenesis as well as changes in nutrient recycling and primary productivity on source rock development. The results also highlight the interrelated nature of the controlling factors with various feedback mechanisms.

## Study area and analytical approach

**Figure 1:**
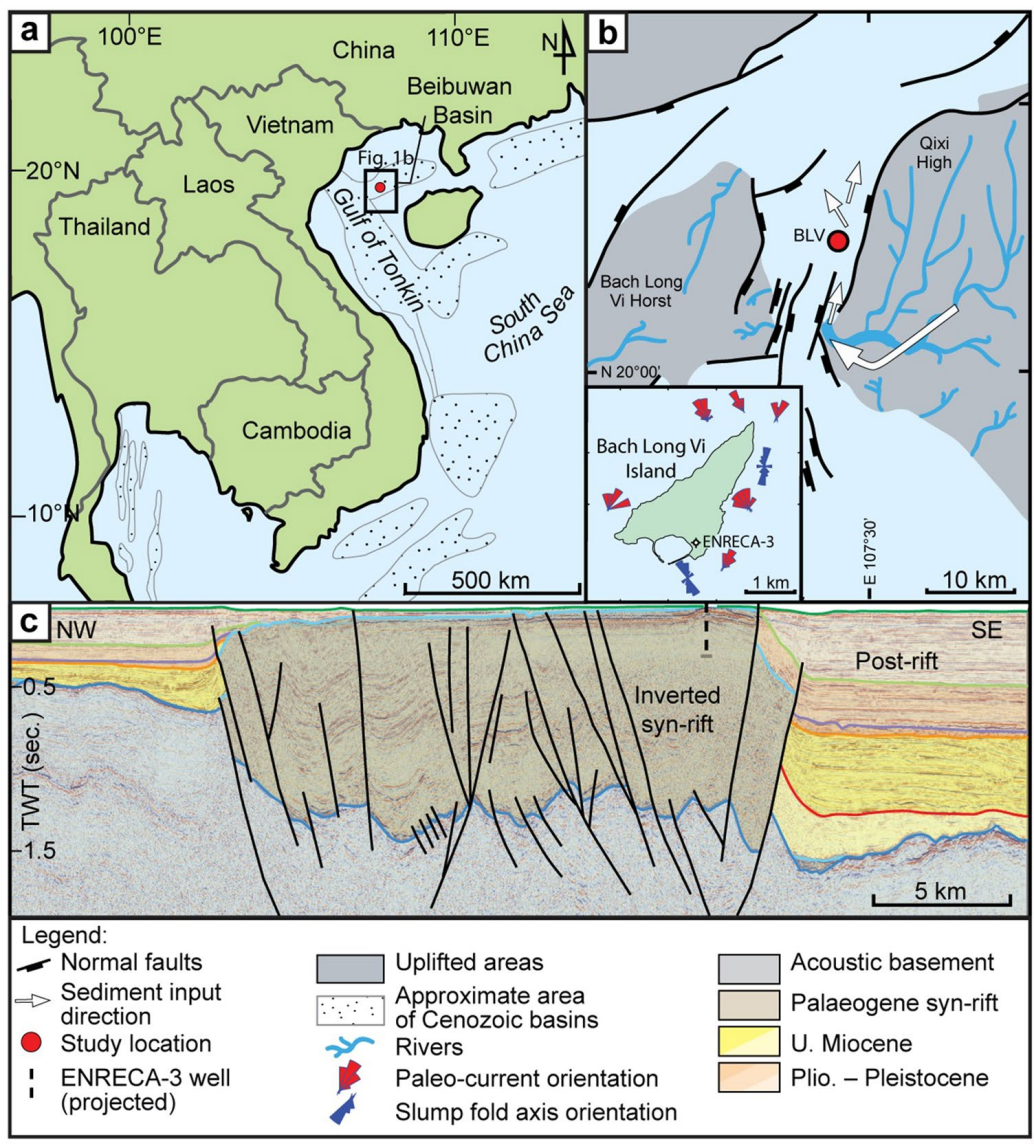
(**a**) Geographical map with outlines of the depositional basins. Both, the core and Bach Long Vi Island are marked with red dot (20° 07′ 39.4″ N, 107° 43′ 49.4″ E). (**b**) Reconstruction of the graben (light blue) with inferred paleohighs (grey) and sediment delivery system (modified from Rizzi et al.^20^). BLV—Bach Long Vi Island. (**c**) An interpreted seismic transect (~ 7 km long) across the Bach Long Vi Graben located near the island (after Fyhn et al.^18^). TWT = two-way time.

The data were collected from a 500 m long drill-core supplemented by observations from the overlying ~ 300 m thick succession that crops out on Bach Long Vi Island, Vietnam (Fig. 1). Previous studies have indicated that the studied deposits represent a deep, freshwater, oil-prone rift lake system^15–18^, deposited during the upper Oligocene^18,19^. The rift basin was relatively sediment starved and intermittently, throughout its existence, received phosphate-rich sediments from a single footwall block^20^.

In brief, HH-XRF Analyser data (*n* = 2,464) were used to derive elemental proxies for depositional environment present in the lake at the time of deposition. Insight into primary productivity was gained by analysis of P/Al and Ba/Al ratios^21–26^. Phosphorus is crucial to organisms, since it has an elementary role in various metabolic processes^24^. With death of the organisms it is released from the decaying organic matter and can be incorporated as precipitates in the sediment. This allows to use the P/Al as a proxy for paleoproductivity. Barite is similarly incorporated by phytoplankton and also form as precipitates in the sediments following organic matter decay (see e.g. Tribovillard et al.^24^). Limitations of these proxies include that accumulation of P is dependent on the redox conditions and that of Ba on organic matter degradation. Water column oxygenation can be inferred from different behavior of redox sensitive elements (e.g. Mn/Fe)^25,27–29^ or by comparison of redox sensitive elements with highly insoluble ones (e.g. V/(V + Ni))^30^. In case of the Mn/Fe ratio, the oxygenation of the water column can be inferred based on the fact that Mn is reduced more rapidly than Fe under anoxic conditions, what leads to preferential Mn release and therefore low Mn/Fe ratios^27,29^. For V/(V + Ni) ratio oxygenation can be inferred, since V is highly soluble in oxic waters but occurs as insoluble precipitate under anoxic conditions while Ni is not affected by redox conditions^26^. Therefore low V/(V + Ni) values are consistent with more oxic environments.

Those proxies are used both in marine and lacustrine settings. However, since most of the threshold ratios of the proxies have been developed for marine settings (e.g. for V/(V + Ni) values between 0.46 and 0.60 point to dysoxic conditions and between 0.54 and 0.82 anoxic conditions^30^), it is reasonable to use only the relative differences between them^31^.

Recognition of excess silica that is consistent with biogenic origin can be inferred from comparison of this element to those of purely detrital origin, such as zircon (Si/Zr)^32,33^.

Strontium/copper (Sr/Cu) ratio has been used as a paleoclimate proxy^34–36^. It has been suggested that values between 1.3 and 5.0 point to a warm-humid climate and those > 5.0 reflecting more a hot-arid climate^34^. This ratio is shown in this study, however since the mechanism description is not fully available in English, the implications are considered as very tentative.

To strengthen the interpretation of the element proxies biomarker data (*n* = 49) were used. The pristane/phytane (Pr/Ph) ratio can be used as a paleoredox indicator^37,38^ since phytane is preferentially forming over pristine in chlorophyll-a under anoxic conditions. Gammacerane serve as sign for water column stratification^39^ since anaerobic ciliates living at the interface between stratified water layers are rich in this component. Therefore, the organic matter derived from those organisms preserves information about paleoredox and stratification conditions.

**Figure 2:**
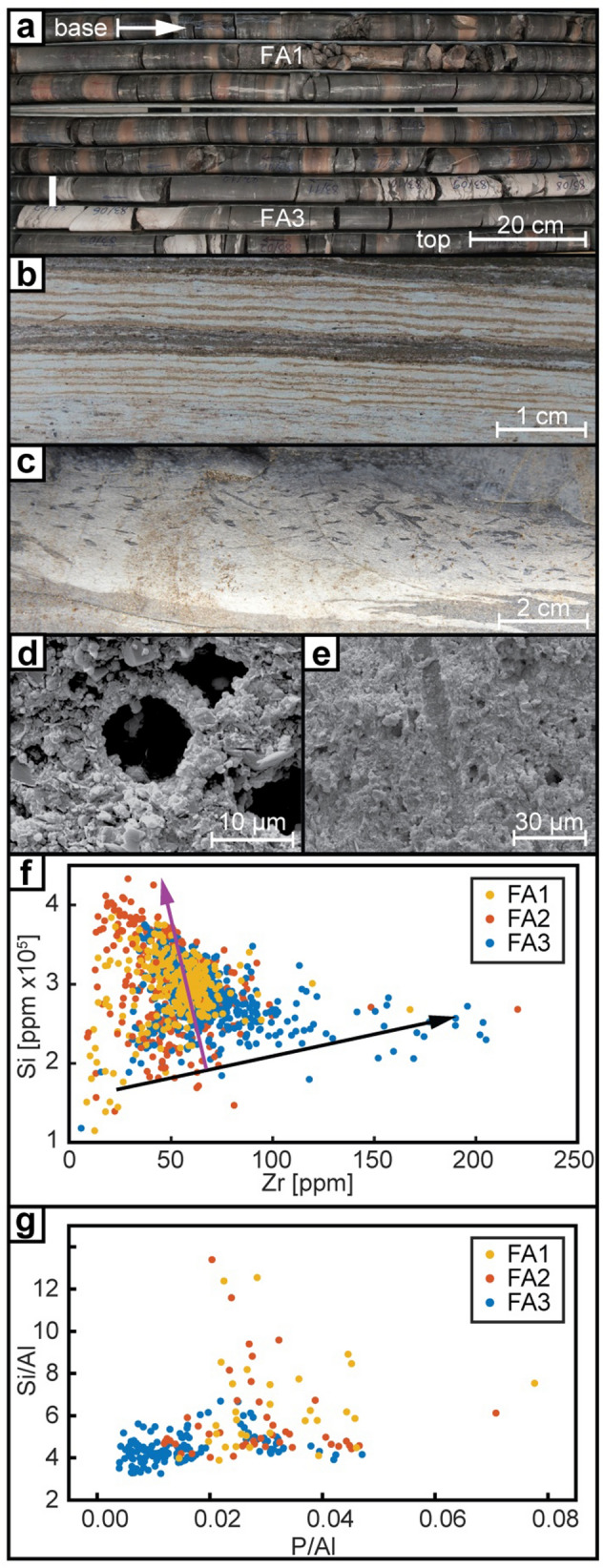
(**a**) Photo of 8 m of the core (410–417 m), showing change from FA 1 to FA 3. (**b**) Thin section photomicrograph of varved sediments from 161.67 m core depth. (**c**) Photo of bioturbated diatomite from the north coast of Bach Long Vi Island. (**d**,**e**) SEM image of dissolution voids after diatom frustules from 161.67 m core depth. (**f**) Cross plot of Si vs. Zr content in different FAs. Black arrow indicates detrital, while purple arrow biogenic silica trends. (**g**) Cross plot of Si/Al vs. P/Al ratios in different FAs. The data were binned in two meters intervals using their mean value.

Total Organic Carbon (TOC) and Hydrogen Index (HI; *n* = 256) data were published previously by Petersen et al.^15^. Additional TOC data (*n* = 1,078) measured on an ELTRA CS-500 Carbon Sulphur apparatus are presented. Other datasets include maceral analyses (*n* = 11), siderite δ^13^C (*n* = 6), SEM photomicrographs (Fig. 2d,e; *n* = 17) and thin sections (Fig. 2b; *n* = 24) that were used to investigate the sedimentological structures in detail.

## Results and interpretation

**Figure 3:**
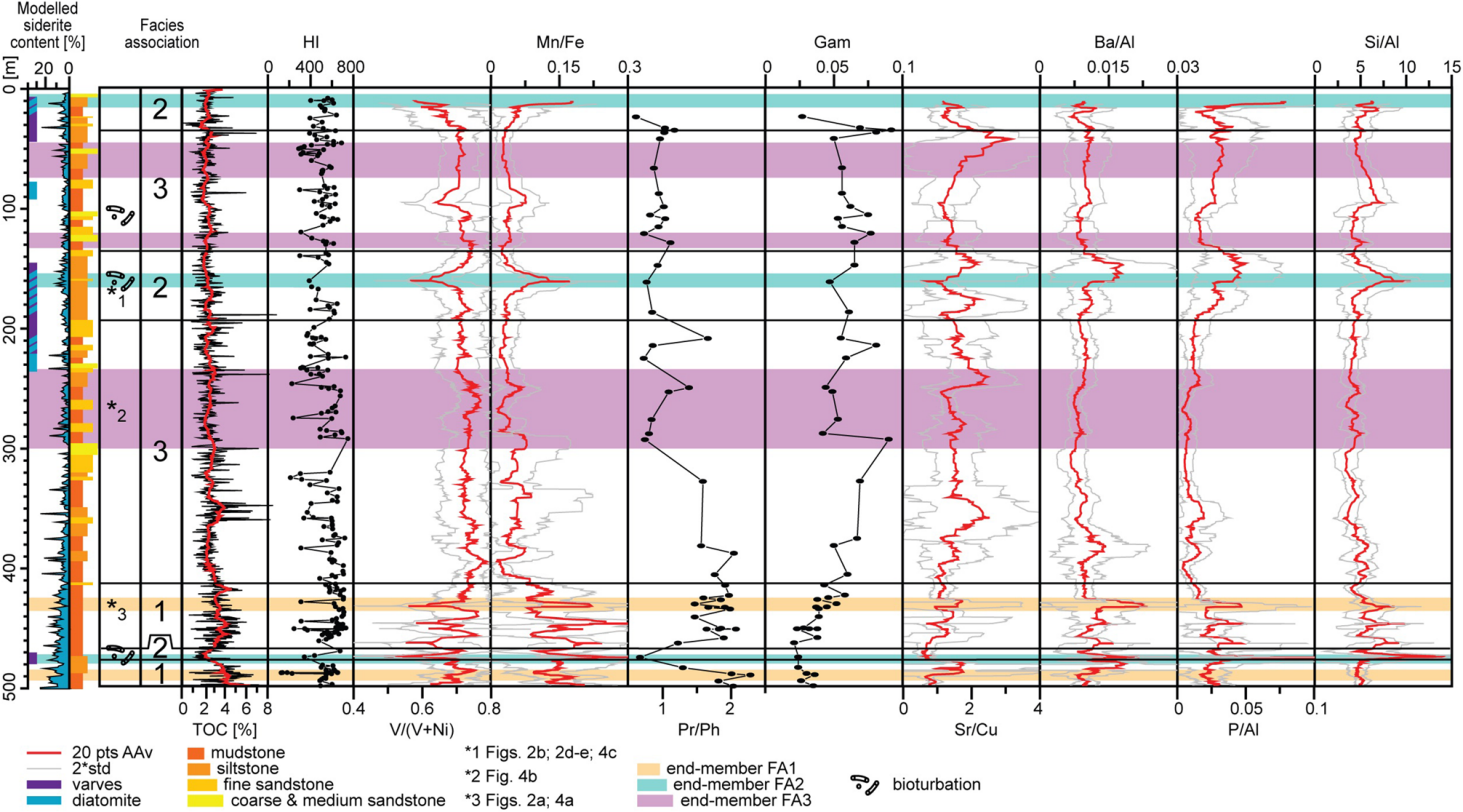
Overview of the element proxies (20 points weighted running average) and biomarkers shown against simplified log of the core. The confidence intervals (grey lines) are set as 2 * std from the mean. On the left side, the modelled siderite content and a representation of sampled lithologies is shown. On the right side, the modelled siderite content, macroscopically recognized varved sediments and diatomite deposits are shown. Black horizontal lines show the FA boundaries.

**Figure 4:**
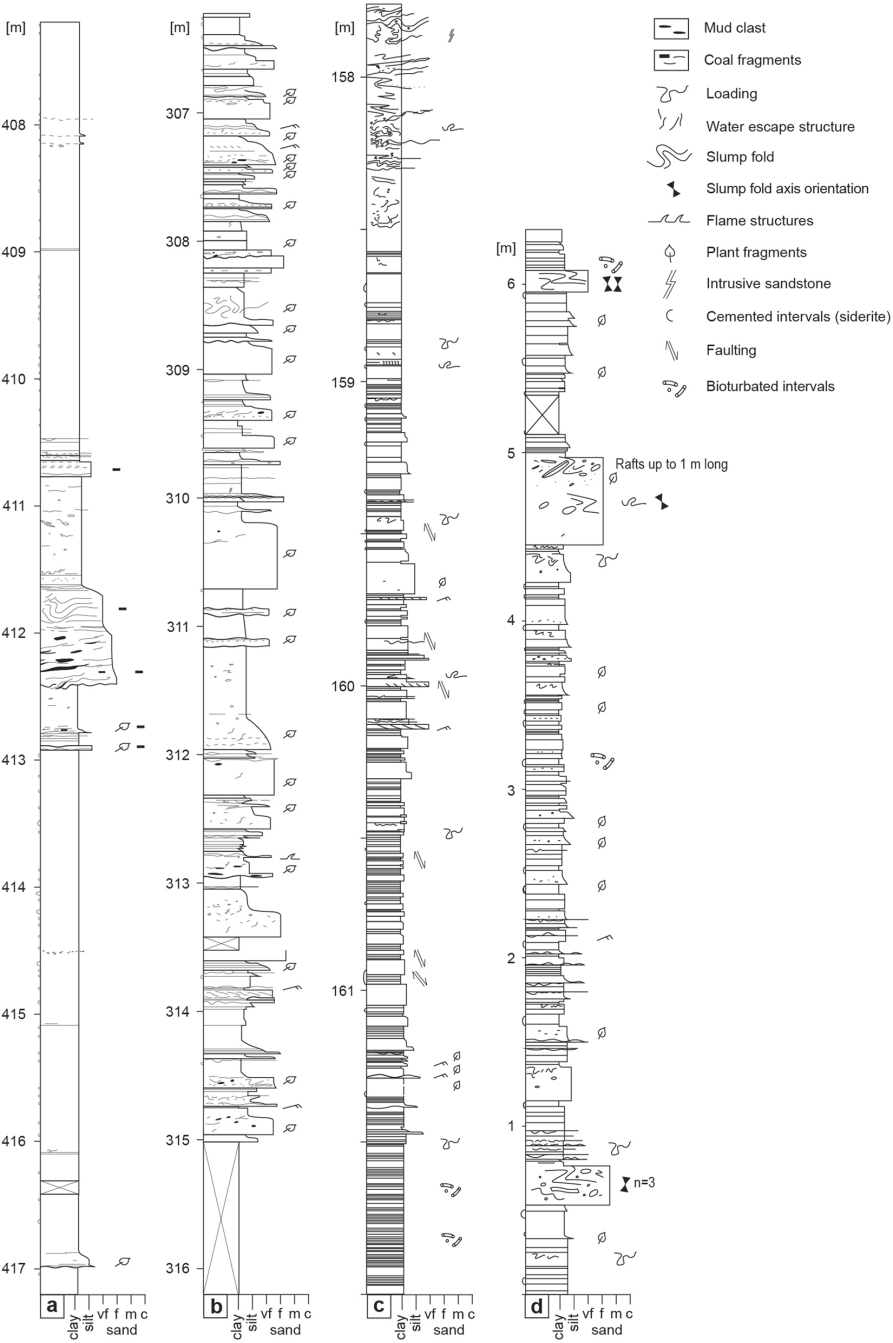
Sedimentological columns from the ENRECA-3 core (**a**–**c**) and the north coast of Bach Long Vi Island (**d**). (**a**) The transition from FA1 to FA3. The log illustrates basinal mudstone intervals that are locally interbedded with decimeter to a meter thick, mud-dominated slurry gravity flow deposits. (**b**) Interval dominated by FA3. The section is characterized by sand-dominated accumulations of turbulent, transitional and laminar flow deposits, which are interbedded with basinal black mudstone intervals. (**c**) The FA2 interval is characterized by mud-dominated transitional plug flow facies interbedded with varve-bearing intervals. Diatomites occur recurrently and are locally bioturbated. The top of the section shows a chaotic interval with common sediment intrusions. (**d**) Interval of FA2 deposits characterized by mud-rich deposits of transitional flows (e.g., fluid muds—low-strength cohesive debrites) and local low-density turbidites, which are interbedded with varve-bearing intervals. Diatomites are commonly well developed. Secondary facies include high-density turbidites, debrites and slump folded beds (transitional intervals to FA3).

The deposits are divided into three facies associations (FA1-3) based on their sedimentological and geochemical characteristics. The data are presented in Table 1 and Figs. 2, 3 and 4, and briefly summarized and interpreted below.

**Table 1:** Summary of facies associations.

Occurence		Sedimentology	Org. chem.	Proxies	Min	Median	Max	Maceral
FA 1	Td–478 m, 469-412 m	The deposits are characterized by weakly laminated to structureless dark grey mudstone, cm-scale concretionary siderite bands and ripple cross-laminated lenses. Fine grained lithologies (mudstone to siltstone) constitute 96% of the sediments, of which siderite mineral constitutes on average 11–15%	Mud	V(V + Ni)	0.61	0.69	0.74	Common lamalginite (avg. 15%), telalginite (avg. 13.7%) and AOM (avg. 13.1%) with secondary vitrinite (avg. 4.5%). Siderite intervals show a relative increase in vitrinite, liptodetrinite mixed with AOM and mineral matter content
			TOC: 3.68 wt% HI: 607 mg HC/g	Mn/Fe	0.09	0.15	0.25	
				Pr/Ph	1.22	1.85	2.30	
				Gam.	0.02	0.04	0.06	
			Siderite	Sr/Cu	0.71	1.07	1.69	
			TOC: 1.1 wt% HI: 212 mg HC/g	Ba/Al	0.01	0.01	0.02	
				P/Al	0.02	0.02	0.04	
				Si/Al	4.39	5.17	8.27	
FA 2	478-468 m, 193-137.5 m, 27-5 m, overlying outcrop interval	The deposits are characterized by pale microlaminated mudstone and mud-dominated gravity flow deposits. Fine-grained lithologies (mudstone to siltstone) constitute 90% of the sediments, of which siderite mineral constitutes on average 4–10%	Mud	V(V + Ni)	0.57	0.66	0.73	An intermediate sample with FA1 (479 m) indicates dominance of AOM (29.6%) with secondary telalginite (10%), lamalginite (4%), and vitrinite (4%)
				Mn/Fe	0.03	0.07	0.22	
			TOC: 2.07 wt% HI: 516 mg HC/g	Pr/Ph	0.61	0.77	1.12	
				Gam.	0.02	0.05	0.07	
				Sr/Cu	0.65	1.26	1.91	
				Ba/Al	0.01	0.01	0.02	
				P/Al	0.02	0.03	0.08	
				Si/Al	4.22	5.66	11.77	
FA 3	412-193 m, 137.5-27 m	The deposits are characterized by black structureless mudstone interbedded with various gravity flow facies. Sand-dominated gravity flow deposits constitute 22% of the sediments. Siderite mineral content in the fine fraction sediments constitutes on average ~ 3%	Mud	V(V + Ni)	0.71	0.73	0.76	Dominance of AOM (avg. 41.9%) with secondary vitrinite (avg. 7.4%) telalginite (avg. 8.6%) and lamalginite (avg. 2.7%)
				Mn/Fe	0.02	0.04	0.07	
			TOC: 2.32 wt% HI: 532 mg HC/g	Pr/Ph	0.72	0.99	1.98	
				Gam.	0.04	0.06	0.09	
				Sr/Cu	1.15	1.49	2.18	
				Ba/Al	0.01	0.01	0.01	
				P/Al	0.01	0.01	0.03	
				Si/Al	3.72	4.33	5.39	

*FA* Facies Associations, *Gam* gammacerane.

### FA1: low-gradient dysoxic lake

Facies Association 1 is a mudstone dominated FA-type, which occurs in the lower part of the core (e.g., Td–478 m, 469‒412 m). It characteristically consists of weakly microlaminated to structureless dark grey mudstone and concretionary siderite bands that together form trendless and aggradational successions (Fig. 2a; Table 1). The microlaminated fabric comprises silt-lamina, which are interlaminated with micritic siderite and disseminated amorphous organic matter (AOM). Apart from local small-scale ripple cross-laminated beds, the sediments typically lack gravity-flow deposits and major indicators of slope instability such as well-developed slump deposits (Fig. 4a; cf. FA3, Fig. 4b). In a few cases, the ripples appear symmetric and show laminae offshoots typical for wave ripples (478.8 m and 465.3 m).

Anoxia proxies are variable, but particularly Pr/Ph (median 1.85) suggests generally dysoxic conditions^37,38^ (Fig. 3). Similarly, the consistently low to absent gammacerane content (median 0.04) points to the lack of a well-stratified water column^39^, compatible with partial mixing of the epi- and hypolimnion. The sedimentary facies, high alginite content (28.7%), and somewhat fluctuating P/Al, Ba/Al and HI (median of 0.02, 0.01 and 607 mg HC/g, respectively) values suggest moderate nutrient recycling and productivity^21–25^, coupled with very limited or lack of direct deltaic sediment input (see “Discussion”). The mudstone sediments in this FA have the highest TOC content (average 3.68 wt%). Finally, δ^13^C data from siderite concretions (from + 6.3 to + 9.3‰ V-PDB) suggest that siderite formation involved methanogenesis^40–42^. In addition, biomarkers from siderite and adjacent black mudstones are essentially similar and show that the amount of terrestrially derived OC is not higher in the siderite intervals. These data indicate that prominent drops in OM quantity (TOC drops from 3.81 to 1.10 wt%) and quality (HI drops from 607 to 212 mg HC/g) are due to methanogenesis related degradation (see Table 1).

### FA2: varve-producing lake

Facies Association 2 is a mudstone-dominated FA-type, occurring in the lower part of the core as an incipient variant (478–468 m). Well-developed occurrences are present in the upper half of the core (5–27 m, 137.5‒193 m; Fig. 4c) and overlying outcrop exposures (Fig. 4d). It is characterized by pale micro-laminated mudstone (Fig. 2b; Table 1) and forms meter-scale aggradational successions.

The microlamination consists of recurring lamina couplets: pale mudstone laminae that comprise illite/kaolinite clay particles and is variably sideritic, and grey lamina which is characterized by abundant microporosity formed by circular to elongate holes typically 5–10 µm in diameter. SEM data show that the voids are rimmed by micro-crystalline quartz (Fig. 2d,e). The XRF derived Si/Zr ratio points to a biogenic origin for silica^32,33^ (Fig. 2f), thus suggesting that the voids are generated by dissolution of diatoms. The tabular micron-scale interlamination and the rarity of pinch-outs, convolute-lamination, scours, lenticularity or base/top lapping lamina contacts in mudstone facies agree with low-energy sediment settling (Fig. 2b; see e.g., Schieber et al.^43^, Macquaker and Bochas^44^). The micro-laminated fabric with alternating clay laminae and inferred diatom-rich laminae suggests regular changes in the lake system from phases of clastic sedimentation to diatom blooms with reduced clastic input. This is consistent with seasonal varve sedimentation.

Secondary facies include cm-scale silty to sandy mudstone beds interpreted as gravity flow deposits^17^. The beds comprise a basal silty mudstone layer commonly overlain by a pale mudstone interval rich in biogenic silica, which is further overlain by a graded mud-drape interpreted as suspension fall-out. The silica interval is interpreted as diatomite, and its position in the bed motif indicates that it commonly forms the last part of the gravity flow event and was transported from a shallower part of the lake. Bioturbation is often restricted to this interval demonstrating that the burrowing organism were transported into the deep lake with the gravity flow events and died soon after (Fig. 2c; “doomed pioneers”^45^).

Similar to FA1, FA2 normally lacks sandstone-dominated gravity-flow deposits (cf. FA3). This may point to tectonic quiescence and filling of mainly inherited accommodation space.

The redox proxies are variable, generally suggesting dysoxic to anoxic conditions (V(V + Ni) from 0.57 to 0.73; Pr/Ph from 0.61 to 1.12)^30,37,38^. However, the end-member with the largest concentration of diatomite is associated with a prominent drop of V/(V + Ni) values suggesting slightly higher oxygen levels^30^ (Fig. 3; e.g., ~ 160 m core depth). Similarly, the Mn/Fe ratio (median 0.07) points to increasing oxygenation^28,29^ during deposition of these intervals, and the low gammacerane values (minimum 0.02) suggest a poorly stratified to un-stratified water column^39^. In contrast, the sporadic Pr/Ph values are invariably low (< 1.5) pointing towards a nearly anoxic to anoxic episodes^38^. This is also consistent with bioturbation being most commonly associated with gravity-flow associated diatomites, but is commonly missing from adjacent varve-bearing intervals. The disparity between redox proxies could indicate that anoxia developed intermittently at/below the sediment–water interface due to OM decay, whereas the water column was poorly stratified and commonly became dysoxic. The phosphorous values are variable (P/Al 0.02‒0.08) being in line with fluctuating redox conditions. The elevated Ba/Al values and moderately low P/Al values correlate with elevated silica values (Fig. 2g), which point to increased primary productivity at these intervals. This suggests that bottom anoxia coupled with weaker water column stratification improved nutrient recycling^46^. However, the associated OM is dominated by AOM (29.6%) and the OM quantity and quality is lower than in the other FAs (2.07 wt% for TOC and 516 mg HC/g for HI). This could reflect potential dilution of the OM due to presence of biogenic silica, slight oxidation or diagenesis of the sediments.

### FA3: tectonically active anoxic lake

Facies Association 3 consists of sandstone-mudstone lithologies and forms the dominant FA-type in the middle and upper half of the core (~ 412–193 and 137.5–27 m). It is characterized by black, structureless, sapropelic basinal mudstone interbedded with various sand-prone gravity-flow facies^17^. The deposits include low and high density turbidites, transitional flow deposits and debrites^17^. The deposits are locally arranged into hybrid event beds. In addition, slump folds, sand and mud intrusions, loading- and water-escape structures occur recurrently.

Gravity-flow dominated intervals can form trendless, upward coarsening or fining intervals a few m thick (Fig. 4b). The change from FA1 to FA3 at ~ 412 m is gradational and defined by the appearance of sand-intrusions and up to a meter thick, sharp based and normally graded mud-dominated beds interpreted as gravity-flow deposits (Figs. 2a, 4a). In view of the appearance of slumps and cohesive debrites, FA3 is interpreted to represent a higher gradient depositional system (see “Discussion”).

The anoxia proxies V/(V + Ni), Mn/Fe and Pr/Ph (median 0.73, 0.04 and 0.99, respectively) all point to anoxic or near-anoxic conditions^27–30^ (Fig. 3). Similarly, the gammacerane content (from 0.04 to 0.09) fluctuates, but reaches the highest values of the studied succession suggesting stronger water column stratification relative to other lake phases^39^. Bioturbation is normally absent, even in gravity-flow facies, which is in line with the geochemical proxies. Phosphorous and barium values are invariably low (median P/Al = 0.01 and B/Al = 0.01), suggesting that despite remobilization of P in the anoxic lake bottom, the nutrient recycling from the hypoliminion to the epilimnion was limited. Organic matter in this interval is dominated by AOM (41.9%), while the TOC content and HI values are intermediate in comparison to other FAs (2.32 wt% for TOC and 532 mg HC/g for HI).

## Discussion and conclusions

Previous studies have indicated that the deposits represent syn-rift lake sediments and that the main border fault was located a few km to the East from the study locality^47^ (Fig. 1b). Seismic data show that the investigated Bach Long Vi Graben outlines a narrow, 5‒13 km wide, roughly NNE-SSW oriented sub-basin connected with wider rift depressions in both the north and the south (Fig. 1c). Strong deformation associated with Neogene inversion hampers seismic based analyses of sediment transport directions and gross-depositional facies. However, slump-fold axis orientation and cross-lamination based paleocurrent measurements, combined with the geochemical and seismic data, suggest that the provenance was stable and that the sediments were delivered from a relay-ramp sediment entry point at the southern end of the graben, and that a secondary, nearby sediment source probably existed along the border fault towards the East^20^ (Fig. 1b). During the deep syn-rift lake phases, a steep slope gradient must have existed between the study area and the footwall, making the study area sensitive for slope instability, slope gradient changes and seismic shocks.

In the following discussion, the role of tectonic and climatic factors controlling anoxia are first discussed. This is followed by a summary of lake-phases and factors controlling the OC and SR accumulation in the studied lake system.

The redox proxy data (V/(V + Ni), Mn/Fe, gammacerane, mostly Pr/Ph) suggest that the FA1 and FA2 occurrences at base of the core contain more oxygenated intervals and less stratified water columns^24,25,27–30,38,39^ (e.g., core intervals 483–463 m, 452–447 m) than FA3, which dominates rest of the core (Fig. 3). This interpretation is supported by occurrences of bioturbation (e.g., 470.45 m, 452.0 m, 446.4 m; Fig. 2c), which are limited to these and similar FA2 intervals elsewhere in the core (e.g., 162‒161 m; Fig. 4c) and outcrop (Fig. 4d). Furthermore, the base of the core contains the only observed occurrences of wave ripple cross-lamination (478.8 m, 465.5 m), suggesting a shallower water depth and a lake offshore setting where maximum wave base intermittently reached the lake bottom. Finally, the core interval lacks major gravity flow deposits and indicators of slope instability.

The redox proxies also suggest an upwards change towards a more permanent anoxia^29,30^ starting from ~ 430 m (Fig. 3). Biomarkers (Pr/Ph and gammacerane) suggest that the trend intensifies upwards until ~ 300 m below the surface^38,39^. Tectonic factors such as faulting, evolution of rift basin morphology and consequent decrease in width/depth ratio are considered the primary factors causing this major environmental change in the lake system^10^. The interpretation is based on the following observations. Firstly, the initial change towards anoxia is associated with a facies change characterized by up to meter scale mud-rich, slurry gravity-flow deposits (Fig. 2a), slumps, small-scale mud- and sand-intrusions and loading structures. The interval ~ 300 to  ~ 200 m where proxies suggest dominantly near anoxic or anoxic setting, is characterized by lack of bioturbation, low- to moderate-strength cohesive debrites^48^, several m-thick turbidite beds, pervasive slump folding, loading and sediment intrusions. These data are consistent with slope instability and gradient change, and potentially with earthquake generated liquefaction and fluidization.

Secondly, source rock screening data combined with sedimentology indicate that many gravity-flow beds represent single flow events that intermittently interrupted basinal sapropelic mud accumulation (Fig. 4). Even most of the muddy gravity-flow deposits have SR potential (HI ~ 300‒650), which together with the inferred low event frequency further points to the fact that they represent collapsed lake slope mud rather than direct deltaic gravity flow sedimentation^17^.

Thirdly, the increase in anoxia is mainly unrelated to changes in the putative Sr/Cu climate proxy in this interval (430‒300 m). However, Sr/Cu does correlate locally with the V/(V + Ni) and can have an inverse correlation with Mn/Fe, and Si values elsewhere in the core (Fig. 3; FA2: ~ 475 m, ~ 160 m). Tentatively, this could suggest that relatively hotter intervals are linked to increasing water column deoxygenation, increased thermocline and reduced nutrient recycling to epilimnion, lower biogenic silica values, whereas a cooler climate may be associated with opposite trends. Thus, climatic forcing may potentially be an important factor in the change from non-siliceous sapropelic sedimentation (FA3) to diatom blooming (FA2). Furthermore, given that diatomites are associated with varve sediments, the water column mixing was likely due to seasonal overturn.

Although the sampling frequency in the present data set is not ideally suited for detecting high-frequency lake level changes, the data indicate a general, sapropel-rich offshore to deep lake setting throughout the 800 m thick study interval. Lyons et al.^11^ reported significant reduction in TOC values (from 5 to 0.2%) during major lake level lowstands in the Lake Malawi. Despite of some scatter in TOC, HI and XRF-derived elemental data values in the present study (Fig. 3; Table 1), the environmental variability does not seem as dramatic as the Quaternary lake level fluctuations in East Africa. Since climate in the region during the late Oligocene was relatively stable, cool to warm and mostly seasonal^49^ the high-frequency climatically induced changes in lake level and hinterland vegetation were probably minor and likely to be masked by other factors. This tentative interpretation need to be tested in future work, and require cm-to mm-scale sampling densities across selected intervals. Further investigation regarding the precise age and the depositional rate of the succession is necessary in order to understand if the observed climatic changes were driven by Milankovitch forcing.

Previous studies have shown that OC and SR accumulation are controlled by the complex interplay of primary productivity, dilution and preservation of OM, which are further influenced by a number of factors such as lake level fluctuations, changes in hinterland and basin slope angles and inorganic sediment and terrestrial OM input^3,6,7,10–12,50–54^. Despite recent progress in understanding these processes (e.g., Bohacs et al.^55^, Ellis et al.^6^), SR accumulation in rift-lake systems is particularly complex and therefore remains hard to generalize^3^.

**Figure 5:**
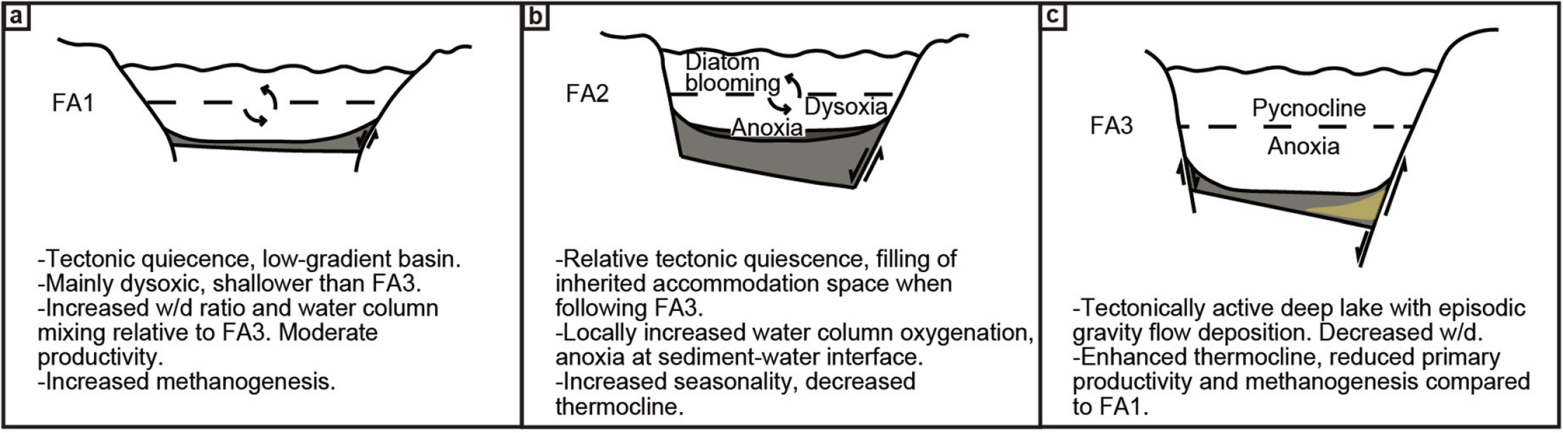
(**a**–**c**) Schematic cross-sections of the different facies associations illustrating interpreted end-members. *w/d* width/depth.

Figure 5 illustrates end-member models of the different depositional scenarios and factors controlling OC and SR accumulation in the studied rift lake. As mentioned above, the data suggest that most of the study interval is generally dysoxic–anoxic, with temporal excursions towards relatively higher oxygen levels in FA2. The dysoxic intervals (e.g., FA1 with Pr/Ph ~ 1.8) contain the richest SR, whereas anoxic or near anoxic intervals^38^ (Pr/Ph ~ 1.2) are more typically associated with slightly reduced TOC values (FA3). Consequently, oxidation of the OM (in dysoxic settings) does not impose a major degrading effect on SR quality in the studied sediments, which is in line with results of several previous studies (e.g. Katz^9^, Lehmann et al.^56^, Harris et al.^57^, Ellis et al.^6^). This interpretation is also supported by the consistently high HI values at times when the SR was unaffected by methanogenesis, excessive detrital sedimentation or terrestrial OM input.

Increased recycling of nutrients and elevated primary productivity generally had a limited impact on source rock quality in the studied lake system. In fact, several intervals with evidence for elevated nutrient recycling and primary productivity are associated with a change from non-siliceous sapropel to diatom-rich sedimentation, characterized by increased biogenic silica and somewhat reduced TOC and HI values (FA2; cf. Bohacs et al.^55^). However, moderately high primary productivity during sapropelic sedimentation had a positive impact on source potential (FA1).

The data also suggest that water column stratification had an important role in nutrient recycling. This is observed in the aforementioned change from FA1 to FA3, where a drop of TOC values broadly correlates with decreasing P and Ba values, and increasing gammacerane values. This suggests that, despite P was increasingly remobilized in the anoxic bottom waters, the enhanced thermocline probably decreased nutrient recycling from the hypolimnion to the epilimnion, and primary productivity. Nevertheless, even the intervals where proxy data point to most limited nutrient remobilization (e.g., 475 m, ~ 20 m) or strongest pycnocline (e.g., ~ 300 m) produce rich SR, suggesting that external nutrient input was sufficient for sapropel formation. Recently, Rizzi et al.^20^ documented that the hinterland sourcing the lake basin comprised phosphate-rich metasediments and granites, which resulted in high input of this nutrient into the graben stimulating primary production and source rock development in the rift lake.

The data also suggest that one of the main SR degrading mechanisms is methanogenesis that resulted in siderite formation in the studied lake system. The siderite content is highest in the lowermost part of the section (Fig. 3; FA1), where it is present in the form of siderite bands. This, together with δ^13^C data from siderite concretions indicate that they formed entirely due to methanogenesis within the methanogenesis zone^42,58^. In this interval, the OM content is highest and anoxia proxies point mostly to dysoxic conditions. Due to the lack of gravity-flow deposits, the slowest bulk sedimentation rates are expected in this interval. These conditions most likely favored siderite formation by promoting methanogenic reactions beneath the lake floor through a combination of: (1) longer sediment residence time near the lake floor allowing for immobilization of the redox boundary within the sediment^59,60^; (2) slightly higher oxygen levels in the bottom waters and therefore higher availability of reactive iron ions^61^; (3) greater availability of OM allowing for development of anoxic conditions in the sediment due to OM decay^41^ and/or by shifting the redox boundary closer to the surface^62^.

In FA1, siderite-rich intervals (siderite mineral content 36‒58%) show reduced HI values relative to the non-sideritic mudstone on average from ~ 600 to ~ 200. Overall, siderite mineral constitutes 11‒15%, 4‒10% and ~ 3% of basinal deposits in FA1, FA2 and FA3, respectively. Considering that OM degrading siderite-band formation can require as little as ~ 30% siderite minerals, the amount of the siderite bands is roughly three times greater than the pure siderite mineral content. This is in line with visual observations of the core that show nearly 40% of the sediments in FA1 are affected by siderite formation (Fig. 2a). The lower siderite mineral content in FA2 and FA3 was most likely caused by lower oxygen levels and OM content and higher sedimentation rates that most likely limited methanogenesis in the sediments.

The result suggesting that methanogenesis acted as a main OC and SR degrading mechanism supplements results derived from modern rift lakes (e.g., Ellis et al.^6^), in which effects of diagenesis on OC accumulation can be difficult to evaluate due to time-scales differences. Essentially, estimating long-term carbon budgets of lake systems relies on the ability to unravel water column and intra-sediment methanogenesis and methane release. This is of importance not only for understanding petroleum SR development, but also for understanding lake systems as sources or sinks of greenhouse gases. This theme requires attention in future studies to test its role in other ancient lake basins.

## Methods

Along the 500 m long ENRECA-3 core 2,464 major and trace elements determinations by hand-held Niton XL3t GOLDD + XRF Analyser (HH-XRF) was made directly on thoroughly brushed rock surfaces that did not show signs of weathering. The HH-XRF device is equipped with an Ag anode and measures at 6‒50 kV and 0–200 µA max and provides semi-quantitative element concentrations. Measurements were done on average every 20–30 cm in order to represent all of the lithologies, however for calculations of the proxies only data from macroscopically identified mudstones and silty mudstones were used. This reduced the dataset to 1,228 data points. Measurements were made for 150 s applying all-geo filtering on 4 different ranges (main—60 s, low—30 s, high—30 s and light—30 s) that allowed for determination of the content of 32 elements. The measurement parameters were selected in order to minimize the error of single elements concentration and to provide the best possible accuracy/measurement-time ratio. Measurements on flat rock surfaces were prioritized over cylindrical core surfaces although the curvature of the core (diameter 5‒10 cm) provide equally good surface to measure on. Standards were run every 10–20 samples and measurements of 5 in-house standards were made on average 4 times per day to allow for drift correction if present. The drift along the day and in between days was found to be negligible. The XRF measuring area is a window approximately 1 cm^2^, therefore, in some regions of the core, where lithology changes even on mm scale, the elemental values represent an average composition of the sediment. Running averages with a 20 point window were calculated on dataset with removed outliers, defined as points that had values higher than mean + 2 * std (where mean and standard deviation (std) were calculated in a 20 point window around each point). The minimum and maximum values of the proxies in Table 1 were calculated on the same dataset and are 10% and 90% percentile of the 20 points running average, while the median is 50% percentile. Those proxies are used both in marine and lacustrine settings. However, since most of the threshold ratios of the proxies have been developed for marine settings, it is reasonable to use only the relative differences between them^31^.

A total of 213 samples from mudstone intervals were collected from the full length of the core for TOC and source rock screening analysis. Those data have been previously published by Petersen et al.^15^.

In short, the samples analyzed by Petersen et al.^15^ at GEUS were crushed to fraction < 250 μm prior to all analyses. The total organic carbon (TOC, wt%) content was determined by combustion of ~ 50 mg of previously decarbonated sample (by HCl treatment) in a LECO CS-200 induction furnace.

To determine the S1 (free hydrocarbons in the sample); S2 (hydrocarbons generated by decomposition of the kerogen) and T_max_ (temperature at maximum S2 generation) ~ 100 mg of sample was pyrolysis in Humble Instruments and Services Source Rock Analyzer (SRA) system. To ensure correspondence to standard Rock–Eval data, the (SRA) instrument was calibrated against the IFP160000 standard. Based on the results the Hydrogen Index (HI = (S2/TOC) × 100) was calculated.

The rest of the TOC were measured on decarbonated powdered material from 1,078 samples using an ELTRA CS-500 Carbon Sulphur apparatus. Samples were combusted for 90 s in 1,350 °C in an oxidizing atmosphere. In-house C and S standard was measured every 10 samples. Drift was found to be negligible and no correction was made. Standard deviation on standard runs was 0.07 for C.

To investigate the sedimentological structures, a total of 30 polished thin sections were prepared and studied by transmitted light microscopy. Furthermore, 17 representative samples were investigated using a Philips SL-40 Scanning Electron Microscope (SEM) at GEUS.

Modelling of the siderite mineral content based on HH-XRF element concentration was using partial least square (PLS) regression model that allows direct correlations to be modelled between y and the multivariate x data, compensating for debilitating co-linearity between x-variables^63^. The modelling was done based on 34 samples, where both XRD and HH-XRF measurements were made. XRD measurements were performed on material powdered from about 4 cm of the core. Multiple XRF measurements were made in the corresponding region of the core. For modelling purposes for each XRD data point average value of the XRF measurements was calculated. The Unscrambler X software was used to perform the multivariate data analysis. The data set was divided in a training set and a test set (32 points each) to control for overfitting, using systematic cross validation on randomly selected data points. The concentration (*c*) of each element measured by XRF was normalized as *c*_norm_ = (*c* −  < *c* >)*/*std(*c*) where < *c* > and std(*c*) are the mean value and the standard deviation of *c* from all the measurements. This was done in order to facilitate the fitting algorithm. For modelling data points lying outside the 3*std from the 20 pts running mean were defined as outliers and removed from the data set.

Mineral composition for the PLS modelling was determined through X-ray diffraction (XRD) on powders obtained from 34 samples. The analyses were made on Bruker-AXS powder diffractometer D8 Advance apparatus, while the mineral composition was calculated using the Rietveld XRD technique^64^.

Lithological representation of the core sediments shows the percentage of occurring sediments in the HH-XRF measurement points binned every 1 m (corresponding to around 4‒5 samples).

For maceral analyses nine black mudstone samples combined with two samples previously reported by Petersen et al.^15^ were used. Sample preparation and analysis are the same as described by Petersen et al.^15^. The core samples were lightly crushed and sieved between 63 μm and 1 mm, and appropriate analysis fractions were embedded in epoxy. Maceral counting by reflected light microscopy was conducted on the ground and polished epoxy pellets using a Leica DMR (new samples) and a Zeiss microscope. The samples were examined in oil immersion using incident white light and fluorescence-inducing blue light. Identification of huminite, inertinite and liptinite macerals followed the descriptions outlined by Hutton^65^, Taylor et al.^66^, ICCP^67^ and Sýkorová et al.^68^. Fluorescing amorphous organic matter (fluorescing AOM) intimately associated with the mineral matrix was counted as part of the liptinite group. A total of 500 macerals and minerals were counted in each sample.

Gammacerane/17α(H),21β(H)-hopane and pristane/phytane ratios were analyzed in 49 source rock samples. The samples are part of a comprehensive biomarker study that is currently in preparation^69^. Samples with elevated terrestrial input are omitted (oleanane/17α(H),21β(H)-hopane > 0.4) from the present data set to only compare basinal facies with similar organic matter content. All analyzed samples are thermally immature (average Tmax 431 °C, vitrinite reflectance 0.36–0.41% Ro; Petersen et al.^15^). Source rocks were extracted with CH2Cl2/MeOH (93:7 v/v) using a Soxtec system. The asphaltenes were precipitated by addition of a 40-fold excess of *n*-pentane followed by resuspension in *n*-pentane and centrifugation (3‒4 times). Saturated hydrocarbons, aromatic hydrocarbons and polar compounds were isolated using medium pressure liquid chromatography^70^. An aliquot of a typical aromatic fraction (2,012,030–22,960; 495.66 m) was separated into 7 subfractions using MPLC according to polarity in order to get cleaner mass spectra of the aromatic compounds.

Gas chromatography of saturated hydrocarbons was performed using a Shimadzu GC-2010 instrument and a ZB-1 capillary column (25 m × 0.25 mm i.d., film thickness 0.10 μm). The temperature program was 5 °C/min from 80 to 300 °C, followed by 15 min at 300 °C. Only a few of the samples were analyzed using GC since long chain *n*-alkanes were obscured by the more abundant pentacyclic triterpanes preventing quantification. Peak areas from GC–MS (*m/z* 71) were used instead. The two methods give slightly different relative response factors for pristane, phytane, *n*-C17 and *n*-C18. Ratios involving these compounds were adjusted accordingly to GC-FID response. Gas chromatography-mass spectrometry (GC–MS and GC–MS/MS) was carried out using an Agilent 6890 N gas chromatograph connected to a Waters (Micromass) Quattro Micro GC tandem quadrupole mass spectrometer. An Agilent HP-5 or Phenomenex ZB-5 column (30 m × 0.25 mm i.d., film thickness 0.10 μm) was used. Samples were analyzed in splitless injection mode. The injection temperature was 70 °C (2 min hold). The temperature program was 30 °C/min from 70 to 100 °C and 4 °C /min from 100 to 308 °C followed by 8 min at 308 °C. The saturated hydrocarbons were analyzed in Selected Ion Monitoring (SIM) and Multiple Reaction Monitoring (MRM) mode with argon as the collision gas. All 62 aromatic fractions and 11 of the saturated fractions were analyzed in full scan mode.

For stable isotope analysis of siderite the sample material was ground in agate mortar and equivalent of 10 mg of carbonate was reacted with anhydrous phosphoric acid in vacuo for 96 h at a constant 100C. The CO_2_ liberated was separated from water vapor under vacuum and collected for analysis. Measurements were made on a VG Optima mass spectrometer. Overall analytical reproducibility for these samples is < 0.1‰ for δ^13^C and δ^18^O (1σ). Data reported is Craig^71^ corrected and converted to solid siderite value using a fractionation factor of 0.00881.

Isotope values (δ^13^C, δ^18^O) are reported as per mil (‰) deviations of the isotopic ratios^72^ (^13^C/^12^C, ^18^O/^16^O) calculated to the V-PDB scale using a within-run laboratory standard calibrated against NBS standards.

## Data Availability

The datasets generated during the current study are not publicly available due to lack of permission to publish the data but are available from the corresponding author on reasonable request.
